# The harmful algae, *Cochlodinium polykrikoides *and *Aureococcus anophagefferens*, elicit stronger transcriptomic and mortality response in larval bivalves (*Argopecten irradians*) than climate change stressors

**DOI:** 10.1002/ece3.5100

**Published:** 2019-04-05

**Authors:** Andrew W. Griffith, Matthew J. Harke, Elizabeth DePasquale, Dianna L. Berry, Christopher J. Gobler

**Affiliations:** ^1^ School of Marine and Atmospheric Sciences Stony Brook University Southampton New York; ^2^ Department of Biological Sciences University of Southern California Los Angeles California; ^3^ Lamont‐Doherty Earth Observatory Columbia University Palisades New York

**Keywords:** *Aureococcus anophagefferens*, climate change, *Cochlodinium polykrikoides*, *Margalefidinium polykrikoides*, ocean acidification, ocean warming

## Abstract

Global ocean change threatens marine life, yet a mechanistic understanding of how organisms are affected by specific stressors is poorly understood. Here, we identify and compare the unique and common transcriptomic responses of an organism experiencing widespread fisheries declines, *Argopecten irradians* (bay scallop) exposed to multiple stressors including high *p*CO_2_, elevated temperature, and two species of harmful algae, *Cochlodinium* (aka *Margalefidinium*) *polykrikoides* and *Aureococcus anophagefferens* using high‐throughput sequencing (RNA‐seq). After 48 hr of exposure, scallop transcriptomes revealed distinct expression profiles with larvae exposed to harmful algae (*C. polykrikoides* and *A. anophagefferens*) displaying broader responses in terms of significantly and differentially expressed (DE) transcripts (44,922 and 4,973; respectively) than larvae exposed to low pH or elevated temperature (559 and 467; respectively). Patterns of expression between larvae exposed to each harmful algal treatment were, however, strikingly different with larvae exposed to *A. anophagefferens *displaying large, significant declines in the expression of transcripts (*n = *3,615; 87% of DE transcripts) whereas exposure to *C. polykrikoides* increased the abundance of transcripts, more than all other treatments combined (*n = *43,668; 97% of DE transcripts). Larvae exposed to each stressor up‐regulated a common set of 21 genes associated with protein synthesis, cellular metabolism, shell growth, and membrane transport. Larvae exposed to *C. polykrikoides* displayed large increases in antioxidant‐associated transcripts, whereas acidification‐exposed larvae increased abundance of transcripts associated with shell formation. After 10 days of exposure, each harmful algae caused declines in survival that were significantly greater than all other treatments. Collectively, this study reveals the common and unique transcriptional responses of bivalve larvae to stressors that promote population declines within coastal zones, providing insight into the means by which they promote mortality as well as traits possessed by bay scallops that enable potential resistance.

## 1 INTRODUCTION

Globally, wild marine fisheries are in decline (Beck et al., 2011; Lotze, 2006; Worm et al., 2009). Overharvest (Jackson, 2001), eutrophication (Nixon, 1995; Officer et al., 1984), disease (Lafferty et al., 2015; Worm et al., 2009), invasion of foreign species (Bax, Williamson, Aguero, Gonzalez, & Geeves, 2003; Stachowicz, 1999), intensifying harmful algal blooms (Gobler et al., 2017), and climate change (Roessig, Woodley, Cech, & Hansen, 2004) have all contributed to large‐scale reductions of multiple commercial fisheries. Of particular concern are lowered abundances of bivalve molluscs (Beck et al., 2011; Worm et al., 2009), including many that provide essential ecosystem services such as improving water clarity (Burkholder & Shumway, 2011; Coen et al., 2007), stabilizing shorelines (Coen et al., 2007), and promoting biodiversity (Abeels, Loh, & Volety, 2012; Tolley & Volety, 2005). Beyond environmental health, shellfisheries (i.e., molluscs and crustaceans) are a significant economic stimulus in coastal zones being valued at ~$50B (USD) globally (FAO, 2016) and are a major source of nutrition (e.g., protein, omega‐3 fatty acids) among many communities (Hibbeln et al., 2007; Smith et al., 2010). Hence, the decline and/or collapse of these fisheries have adverse socioeconomic and ecological implications (Beck et al., 2011; Jackson, 2001; Lotze, 2006).

Bay scallops (*Argopecten irradians*) are a prime example of a collapsing bivalve fishery (Blake & Shumway, 2006; Tettelbach et al., 2015; Tettelbach & Wenczel, 1993). Native to shallow, estuarine habitats along the eastern United States (Blake & Shumway, 2006; Clark, 1965), bay scallop populations have been in decline since the early 1980s, during which US landings have been reduced by ~97% (NOAA Fisheries Landings 1980–2016; www.noaa.gov). While overharvest and habitat loss have contributed to some of these losses (Barber & Davis, 1997; Serveiss, Bowen, Dow, & Valiela, 2004), the emergence of harmful algal blooms (HABs), specifically the brown tide alga, *Aureococcus anophagefferens *(Hoagland, Anderson, Kaoru, & White, 2002; Tettelbach & Wenczel, 1993) during the mid‐1980s nearly extirpated bay scallop populations in NY and other regions of the northeast United States. Recovery of these populations are now hampered by annual *Cochlodinium* (aka *Margalefidinium*) *polykrikoides *blooms (Gobler et al., 2008; Tang & Gobler, 2009a,2009b). Similar die‐offs have been observed in North Carolina where a large bloom of *Karenia brevis *caused a near‐collapse of the fishery during the late‐1980s (Peterson & Summerson, 1992; Summerson & Peterson, 1990). Beyond harmful algae, bay scallops are generally more sensitive to abiotic stressors associated with climate change including elevated temperature (Talmage & Gobler, 2011), acidification (Talmage & Gobler, 2009), and low dissolved oxygen (Clark & Gobler, 2016) than other North Atlantic bivalves (e.g., *Mercenaria mercenaria*, *Crassostrea virginica*, and *Mytilus edulis*). While stock restoration efforts have exhibited some success (Tettelbach et al., 2013), recurring harmful algal blooms and the intensification of climate change may limit this vulnerable fishery further.

Climate change processes are making coastal marine environments warmer (IPCC, 2014; Solomon, 2007), acidified (Doney, Fabry, Feely, & Kleypas, 2009; Doney et al., 2012), increasingly hypoxic (Breitburg et al., 2018; Keeling, Kortzinger, & Gruber, 2010), and host to intensifying harmful algal blooms (Glibert et al., 2014; Gobler et al., 2017). Since the 1900s, ocean temperatures have risen more than 1.5°C while pH levels have decreased ~0.1 units (Doney et al., 2012; Solomon, 2007). Under “business as usual” scenarios (see IPCC, 2014), temperatures will increase an additional 2–5°C (IPCC, 2014; Saba et al., 2016) this century (*ca* 2,100) as pH levels continue to decline (e.g., 0.2–0.3 units; Heogh‐Guldberg et al., 2014). Additionally, ocean warming has expanded the distribution and lengthened the blooming season of several harmful algae within temperate zones of the Northern Hemisphere (Gobler et al., 2017). The organism‐level (e.g., survival, growth, and development) impacts of these stressors have been well‐studied, but less is known regarding their physiological and mechanistic effects. There is a growing body of knowledge regarding the gene‐level responses of organisms exposed to climate change stressors (Chapman et al., 2011; De Wit, Durland, Ventura, & Langdon, 2018; Goncalves et al., 2016; Zhang et al., 2012). Studies of this type provide insight regarding the physiological impacts of climate change on marine life and reveal sensitive biochemical pathways. Recently, De Wit et al. (2018) investigated differential gene responses of Pacific oyster (*Crassostrea gigas*) larvae exposed to acidification and reported a down‐regulation of genes associated with shell growth (e.g., nacrein, papilin, and chitin‐binding proteins), suggesting that low expression of these genes may be associated with slower larval growth in acidified environments. In a similar study, Goncalves et al. (2016) observed larvae originating from adult, Sydney rock oysters (*Saccostrea glomerata*) exposed to acidification during gametogenesis expressed a greater abundance of transcripts encoding antioxidant enzymes, heat shock, and cytoskeletal proteins compared to larvae originating from adults reproductively conditioned at ambient pH. Studies assessing gene‐level responses within marine life exposed to harmful algal blooms have, however, been rare. Further, while the effects of climate change stressors on organism physiology have been described, studies comparing the effects of multiple stressors on biochemical responses have also been rare. While some studies have assessed gene responses among oysters exposed to acidification (*see above*), few have assessed the responses to other stressors or the responses of other commercially and ecologically important bivalves.

Here, we used high‐throughput transcriptomic sequencing (RNA‐Seq) to identify gene expression responses of *A. irradians* (bay scallop) larvae exposed to elevated temperature, acidification, and two species of harmful algae, *A. anophagefferens *and *C. polykrikoides*. Along with identifying pathways uniquely invoked by each stressor, a secondary goal of the study was to assess biochemical pathways commonly expressed in response to all stressors. Collectively, our findings provide insight into the molecular mechanisms by which larvae respond and potentially defend themselves against these stressors and the mechanisms by which they are harmed, information that may aid in the preservation of marine resources in the future.

## 2 MATERIALS AND METHODS

### 2.1 Algal cultures

All algal cultures used in this study were maintained at 21°C on a 12:12 hr light:dark cycle with a light intensity of ~100 µmol quanta m^−1^ s^−1^ and were in kept in exponential growth phase during use in experiments. Cultures of *A. anophagefferens* and *C. polykrikoides* were grown in sterile GSe growth medium (salinity = 32–33; Tang & Gobler, 2009b). An antibiotic solution (1% v/v final concentration, 10,000 I.U. penicillin, 10,000 µg/ml streptomycin, 25 mg/ml, amphotericin B) was included in each vessel to minimize bacterial contamination. Cultures of *Isochrysis* spp. were grown in Guillard's *f*/2 (‐ Si) growth medium (Guillard & Ryther, 1962).

### 2.2 Experimental design

Bay scallop larvae were obtained from the Cornell University Cooperative Extension (Southold, NY) using wild‐collected broodstock from the Peconic Estuary, NY, USA. This is a mesotrophic estuary that experienced blooms of *A. anophagefferens* for a decade (1985–1995; Gobler, Lonsdale, & Boyer, 2005) and has experienced annual blooms of *C. polykrikoides* of varying intensities since 2004 (Gobler et al., 2008; Kudela & Gobler, 2012). The estuary experiences peak temperatures of ~26°C in summer and pH levels generally between 7.9 and 8.1 (SCDHS 1985–2017). Larval‐staged individuals were included in analyses as this particular life‐stage represents a critical bottleneck in the life‐history of the bay scallop and early‐larvae are highly sensitive to environmental stress (Shumway & Parsons, 2011; Talmage & Gobler, 2009).

Within 24 hr of fertilization, larvae were added to 8‐L experimental vessels at a density of 2 × 10^3^ larvae L^−1^. Experiments were performed to simulate the exposure of *A. irradians* larvae to stressors common within present‐day estuaries as well as those predicted to become more pronounced in the future. Five specific treatments were included the following: (a) levels of acidification (pH_T_ ~ 7.7; *p*CO_2_ = 1,400 ppm) already common in eutrophic estuaries (Baumann, Wallace, Tagliaferri, & Gobler, 2015; Waldbusser, Voigt, Bergschneider, Green, & Newell, 2011; Wallace, Baumann, Grear, Aller, & Gobler, 2014) and representative of future ocean conditions (Heogh‐Guldberg et al., 2014); (b) elevated temperatures (30°C) indicative of coastal heat waves in the northeast United States and a common summer temperature in the future (Nixon, Granger, Buckley, Lamont, & Rowell, 2004); (c) bloom concentrations of the brown tide pelagophyte, *A. anophagefferens *(10^6^ cells ml^−1^; Gobler & Sunda, 2012); (d) bloom densities of the ichthyotoxic dinoflagellate, *C. polykrikoides *(Gobler et al., 2008); and (e) a control treatment (e.g., 24°C; pH_T_ ~ 8.0; *p*CO_2_ ~ 400 ppm) in which larvae were fed an ideal diet daily (Helm, Bourne, & Lovatelli, 2004). Treatments were administered in four, replicate 8‐L polyethylene vessels filled with 0.2 μm filtered seawater (salinity = 30) collected from eastern Shinnecock Bay (NY, USA; 40.8845°N, −72.4414°W) and maintained in temperature‐controlled water baths at 24°C. An antibiotic solution (1% v/v final concentration; 10,000 I.U. penicillin, 10,000 µg/ml streptomycin, 25 mg/ml amphotericin B) was included in each vessel to minimize bacterial contamination. Carbonate chemistry within the low pH (pH_T_ ~ 7.7, *p*CO_2_ = 1,400 ppm) treatment was manipulated via the addition of concentrated CO_2_ (5%) gas mixed with ambient air delivered into each experimental vessel using multi‐channel gas proportioners (Cole‐Palmer^®^; Talmage & Gobler, 2010). Larvae within all non‐acidification treatments (control, *A. anophagefferens*, *C. polykrikoides*, and elevated temperature) were gently aerated with ambient air at a rate matching the acidification treatment. To simulate thermal stress, temperatures within water baths were increased to 30°C using electronically controlled Delta Star^®^ heat exchangers that maintained levels within 1% of the targeted range.

Every other day, all larvae from each experimental vessel were carefully passaged onto a 64 μm sieve, rinsed with filtered (0.2 μm) seawater, and enumerated via an inverted microscope coupled with a digital Nikon^®^ camera and image analysis software (NIS Elements imaging software; version 3.22.11) to assess larval survival. After enumeration, larvae were resuspended in clean vessels amended as described above. For harmful algal treatments, fresh aliquots of phytoplankton cultures were added to exposure vessels at concentrations described above after each water change. An antibiotic solution (1% v/v final concentration; *see above*) was included in each vessel to minimize bacterial contamination. After 48 hr, ~7,000 larvae (3.5 L) were carefully removed from each experimental vessel and concentrated onto a 64 μm sieve, transferred to cryovials, flash frozen in liquid nitrogen, and stored at −80°C until RNA extraction (*details below*). The remaining larvae were concentrated as described above and resuspended into filtered (0.2 μm) seawater amended as described above. The experiment persisted for 10 days at which point final quantification of the larvae was performed.

Differences in the survival of bay scallop larvae between treatments were assessed with a one‐way analysis of variance (ANOVA) using R^®^ (www.r-project.org, version 3.2.5) statistical software. When significant differences were detected, pairwise comparisons between treatments were performed using Tukey's honest significant difference test (Tukey HSD). All data were confirmed to display a normal distribution with equal variance using Shapiro–Wilk's and Bartlett's tests, respectively. All results were deemed significant at *α* ≤ 0.05.

Temperature and pH_T_ were measured daily in each experimental vessel using a Durafet III (Honeywell) ion‐sensitive field‐effect transistor‐based (ISFET) solid‐state pH sensor. In addition, daily measurements of dissolved oxygen (DO) were made using a YSI^®^ 5100 Clark‐type electrode and indicated DO levels were always above‐ or near‐saturation in all treatments. Prior to, and upon the completion, of experiments, duplicate water samples from the control and acidification treatments were collected for dissolved inorganic carbon (DIC) analysis. Briefly, water from experimental replicates was carefully transferred to 300 ml glass vials, preserved with a saturated mercuric chloride solution, and sealed until analysis. Levels of DIC from control and low pH treatments were determined using an Environmental Gas Analyzer^®^ (EGM—4, PP systems) after acidification and separation of gas phases using a Liqui‐cel^®^ membrane (Membrana). Concentrations of *p*CO_2_, saturation state (Ω) of aragonite and calcite, and total alkalinity (TA) were calculated from total DIC, pH_T_, temperature, salinity, pressure, phosphate, silicate, and first and second disassociation constants for estuarine waters as per Millero (2010) using CO_2_SYS software (http://cdiac.ornl.gov/ftp/co2sys/). Certified reference material (provided by Andrew Dickson; Scripps Institute of Oceanography) was analyzed prior to and after each analytical run for quality assurance and yielded 100 ± 5% recovery.

### 2.3 RNA extraction and sequencing

Total RNA was extracted after 48 hr exposure from duplicate biological samples pooled from two replicates from each treatment using a Qiagen RNeasy Mini Kit according to manufacturer instructions. Quantity and quality of extracted total RNA were assessed using an Agilent 2100 Bioanalyzer (Agilent, Santa Clara, CA). Libraries were prepared using 500 ng of total RNA with an Illumina Truseq kit and then sequenced on an Illumina HiSeq 2000 at the Columbia Genome Center (NY, USA). These sequence data are deposited in the Sequence Read Archive through the National Center for Biotechnology Information under accession no. SRP159112.

### 2.4 Read processing and de novo assembly

Raw sequence quality was visualized using FastQC then cleaned and trimmed using Trimmomatic version 0.36 (paired‐end mode, 4‐bp‐wide sliding window for quality below 15, minimum length of 50 bp). Sequenced reads from all treatments were digitally normalized according to the Khmer protocol (Brown, Scott, Crusoe, Sheneman, & Rosenthal, 2013) and then a combined assembly of all treatments was conducted using Trinity (Grabherr et al., 2011). Resulting assembled contigs were clustered at 98% sequence identity using CD‐HIT‐EST (Li & Godzik, 2006), and the transcriptome was assessed for completeness with Transrate (Smith‐Unna, Boursnell, Patro, Hibberd, & Kelly, 2016) and BUSCO (Simao, Waterhouse, Ioannidis, Kriventseva, & Zdobnov, 2015).

### 2.5 Differential gene expression

Sequence reads from each treatment and replicate (*n* = 2) were aligned to the combined reference assembly using Bowtie 2 (Langmead & Salzberg, 2012) and counted within RSEM v1.2.19 (Li & Dewey, 2011) using parameters recommend by the RSEM authors. Differential expression between experimental treatments and the control were compared using DESeq2 with an adjusted *p*‐value of ≤0.05 as the statistical cutoff (Love, Huber, & Anders, 2014). Expression levels for genes identified in previous studies to be associated with climate change stressors and/or harmful algal exposure (e.g., heat shock protein, antioxidant self‐defenses, immune functions, energy capture, and calcification) were targeted for discussion.

### 2.6 Annotation and functional enrichment

Functional annotation was conducted using the Trinotate pipeline (Haas et al., 2013) preserving annotation assignments from Swissprot (Boeckmann, 2003). In addition, KEGG (Kyoto Encyclopedia of Genes and Genomes) biochemical pathways for each contig were identified with KEGG Automatic Annotation Server (KAAS) using the partial genome single‐directional best‐hit method (Kanehisa et al., 2006; Moriya, Itoh, Okuda, Yoshizawa, & Kanehisa, 2007). Transcripts receiving KEGG assignments were binned by KEGG module. All annotations discussed in the text should be considered putative. For genes discussed herein, consensus annotations were derived based upon available data. If multiple annotations were detected, only one was reported.

## 3 RESULTS

### 3.1 Larval survival

**Figure 1:**
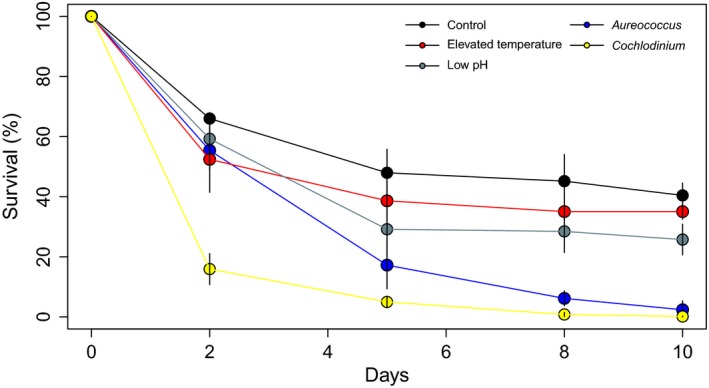
Cumulative survival of bay scallop larvae within each treatment (error bars represent ±*SD*; * denotes significant differences; *p* < 0.05; one‐way ANOVA)

After 48 hr of exposure to all treatments, the time point during which transcriptomic samples were collected, survival of larvae exposed to *C. polykrikoides *was significantly lower (20%) than all other treatments (*p* < 0.05; Tukey HSD; Figure 1). The survival at 48 hr among the remaining treatments was similar (55%–65%; *p* > 0.05; Tukey HSD). After 10‐days, significant reductions in survival were present in the low pH and both harmful algal treatments relative to the control (all *p* < 0.05; one‐way ANOVA; Figure 1) with the survival among acidification‐exposed larvae (e.g., 22 ± 2%, mean ± *SD*; see Table 1 for a summary of carbonate chemistry) being significantly (*p* < 0.05; Tukey HSD) reduced relative to the control and elevated temperature treatments, but significantly greater (both *p* < 0.05; Tukey HSD) than rates among larvae exposed *A. anophagefferens *(3 ± 3%) or *C. polykrikoides* (<1 ± <1%) which did not differ from each other (*p* > 0.05; Figure 1).

**Table 1:** Summary (mean parameter value; *SD* in parenthesis) of dissolved inorganic carbon (∑DIC; µmol kg^−1^), pH, *p*CO_2_ (µatm), carbonate (CO_3_^2−^; µmol kg^−1^), total alkalinity (TA; µmol kg^−1^), saturation state of aragonite (Ω_aragonite_), calcite (Ω_calcite_), temperature (°C), and salinity

Treatment	Low pH	Control
∑DIC	2,102.61 (47.09)	2,018.24 (94.40)
pH	7.69 (0.06)	8.095 (0.03)
pCO2	1,395.55 (182.45)	517.45 (18.87)
Carbonate	68.78 (9.42)	165.53 (25.15)
TA	2,157.43 (9.42)	2,234.58 (124.21)
Ω (aragonite)	1.11 (0.15)	2.68 (0.43)
Ω (calcite)	1.71 (0.24)	4.13 (0.64)
Temperature	24 (0.5)	24 (0.5)
Salinity	30 (1)	30 (1)

### 3.2 Sequencing, assembly, and annotation

Illumina sequencing yielded on average 67 million paired‐end reads, of which 62 million remained after trimming (Supporting Information Table S1). A combined assembly with Trinity produced 753,305 contigs (*N*_50_ = 2,025 bp) which was reduced to 648,465 (*N*_50_ = 1,614 bp) after clustering at 98% identity. Of this clustered set of contigs, 26% contained open reading frames (ORFs; Supporting Information Table S1) and between 80% and 90% of reads aligned to this clustered contig set which were then used for differential expression analysis (Supporting Information Table S2). Annotations from SwissProt revealed information for ~20% of the clustered contigs while KEGG provided assignments to 18% (Supporting Information Table S3).

### 3.3 Differential expression

**Figure 2:**
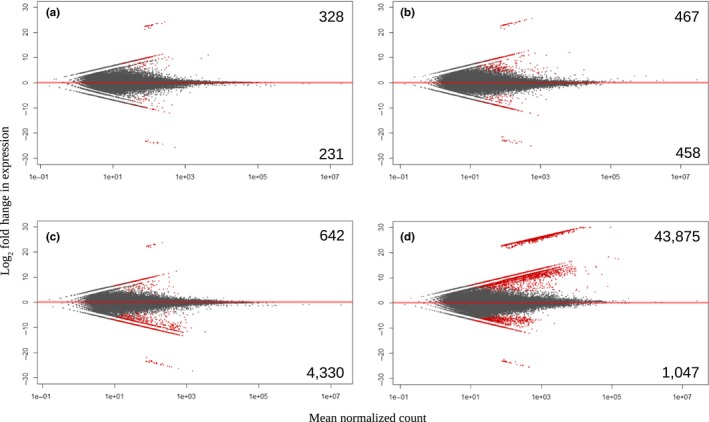
Differential expression (i.e., log fold change) by larvae within low pH (a), elevated temperature (b), *Aureococcus* (c), and *Cochlodinium* treatments (d). Values in the right corners indicate the number of contigs significantly increased (upper) or decreased (lower) relative to the control (red color; *padj* < 0.05)

Exposure to each stressor caused significant (*padj* < 0.05) differences in the expression of numerous transcripts with larvae from each treatment exhibiting a unique transcriptional profile (Figure 2). Larvae exposed to acidification differentially and significantly (*padj* < 0.05) expressed 559 transcripts relative to the control (Figure 2) with 328 of those being present at significantly greater abundance and 231 at lower (Supporting Information Table S4). Transcripts with the highest log_2_ fold change expressed by acidification‐exposed larvae included those encoding proteins associated with heat shock (Hsp), electron transport (cytochrome *c* oxidase I & III), ribosomal‐binding (60s ribosomal protein L5), and shell formation/chitin‐binding (protein obstructor‐E; Table 2). Exposure to thermal stress (e.g., 30°C) stimulated significant (*padj* < 0.05) increases in the abundance of 467 transcripts while suppressing the expression of 458 transcripts. Transcripts encoding phosphatase (serine/threonine‐protein phosphatase) and electron transport (cytochrome *b*, *c* oxidase subunit 1) being among those with the greatest increase in transcript abundance (24.22–24.62 log_2_ fold change; Table 3).

**Table 2:** The 10 transcripts with the largest increases in transcript abundance relative to the control for larvae exposed to acidification

	Fold change (log_2 _fold change)	Protein	Gene ID (Uni Prot)	Function
1	24.31	Heat shock 70 kDa protein cognate 4	—	ATP‐Binding
2	23.70	Apolipophorins	—	Lipid transport
3	23.59	Cytochrome *c* oxidase subunit 3	COIII	Electron transport
4	23.55	Cytochrome *c* oxidase subunit 1	COI	Electron transport
5	23.24	—	—	—
6	23.19	Caveolin‐1	CAV1	Scaffolding protein
7	23.16	60S ribosomal protein L23a	Rpl23a	Ribosome binding
8	23.14	Protein obstructor‐E	Obst‐E	Chitin‐binding
9	22.98	—	—	—
10	22.96	60s ribosomal protein L5	Rpl5	Ribosome‐binding

**Table 3:** The 10 transcripts with the largest increases in transcript abundance relative to the control for larvae exposed to thermal stress

	Fold change (log_2 _fold change)	Protein	Gene ID (Uni Prot)	Function
1	25.72	Serine/threonine‐protein phosphatase 2A 56 kDa regulatory subunit delta‐isoform	PPP2R5D	phosphoprotein phosphatase activity
2	25.17	Outer dense fiber protein 3	odf3	—
3	25.03	Soma ferritin	—	Iron storage
4	24.62	Cytochrome *c* oxidase subunit 1	COI	Electron transport
5	24.54	Myosin heavy chain, striated muscle	—	Muscle contraction
6	24.54	Prostaglandin reductase 2	PTGR2	prostaglandin reductase activity
7	24.46	—	—	—
8	24.37	Cytochrome *b*	MT‐CYB	Electron transport
9	24.30	Cytochrome *c* oxidase subunit 1	COI	Electron transport
10	24.22	Organic cation transporter protein	Orct	Organic ion transport

The number of transcripts differentially expressed among scallop larvae exposed to both harmful algae was one‐ to two‐orders of magnitude greater than the other treatments. For example, larvae exposed to *A. anophagefferens *differentially expressed 4,972 transcripts while exposure to *C. polykrikoides *elicited differential expression of 44,922 transcripts (Figure 2). Of the transcripts differentially (*padj* < 0.05) expressed by larvae exposed to *A. anophagefferens*, a majority (87%) were observed at reduced abundance whereas when exposed to *C. polykrikoides,* 97% of the significantly and differentially expressed transcripts were observed at increased abundance (Figure 2). Transcripts with the largest increases in transcript abundance among both harmful algal treatments included those encoding for RNA‐binding proteins (e.g., protein quaking), phosphatases (e.g., enolase‐phosphatase), and ribosome‐binding proteins (40s ribosomal protein S2 and 60S ribosomal protein L3; Tables 4 and 5).

**Table 4:** The 10 transcripts with the largest increases in transcript abundance relative to the control for larvae exposed to *A. anophagefferens*

	Fold change (log_2 _fold change)	Protein	Gene ID (Uni Prot)	Function
1	23.81	—	—	—
2	23.16	Enolase‐phosphatase E1	ENOPH1	phosphatase activity
3	23.13	—	—	—
4	23.13	Prostaglandin reductase 2	PTGR2	Prostaglandin reductase activity
5	23.08			
6	23.07	Leucine‐rich repeat‐containing protein 74A	LRRC74A	
7	22.75	Protein quaking	Qki	RNA‐binding protein
8	22.74	Hemicentin‐1	Hmcn1	Cytokinesis
9	22.68	Unconventional myosin‐IXb	Myo9b	intracellular movement and growth
10	22.62	Protocadherin Fat 1	FAT1	Cell polarization and cell migration

**Table 5:** The 10 transcripts with the largest increases in transcript abundance relative to the control for larvae exposed to *C. polykrikoides*

	Fold change (log_2 _fold change)	Protein	Gene ID (Uni Prot)	Function
1	30.00	Peptidyl‐prolyl cis‐trans isomerase A	Ppia	Protein folding
2	30.00	NADH‐ubiquinone oxidoreductase chain 1	Mtnd1	Electron transport
3	30.00	Kruppel‐like factor 10	Klf10	Transcriptional repressor
4	30.00	Glyceraldehyde‐3‐phosphate dehydrogenase	Gapdh	Glycolysis
5	30.00	Elongation factor 2	Eef2	Ribosomal translocation
6	30.00	Friend virus susceptibility protein 1	Fv1	Retroviral restriction factor
7	30.00	—	—	—
8	30.00	40S ribosomal protein S2	Rps2	Ribosomal protein
9	29.99	E3 ubiquitin‐protein ligase NEDD4	Nedd4	Ubiquitin‐protein
10	29.91	60S ribosomal protein L3	Rpl3	Ribosomal protein

**Figure 3:**
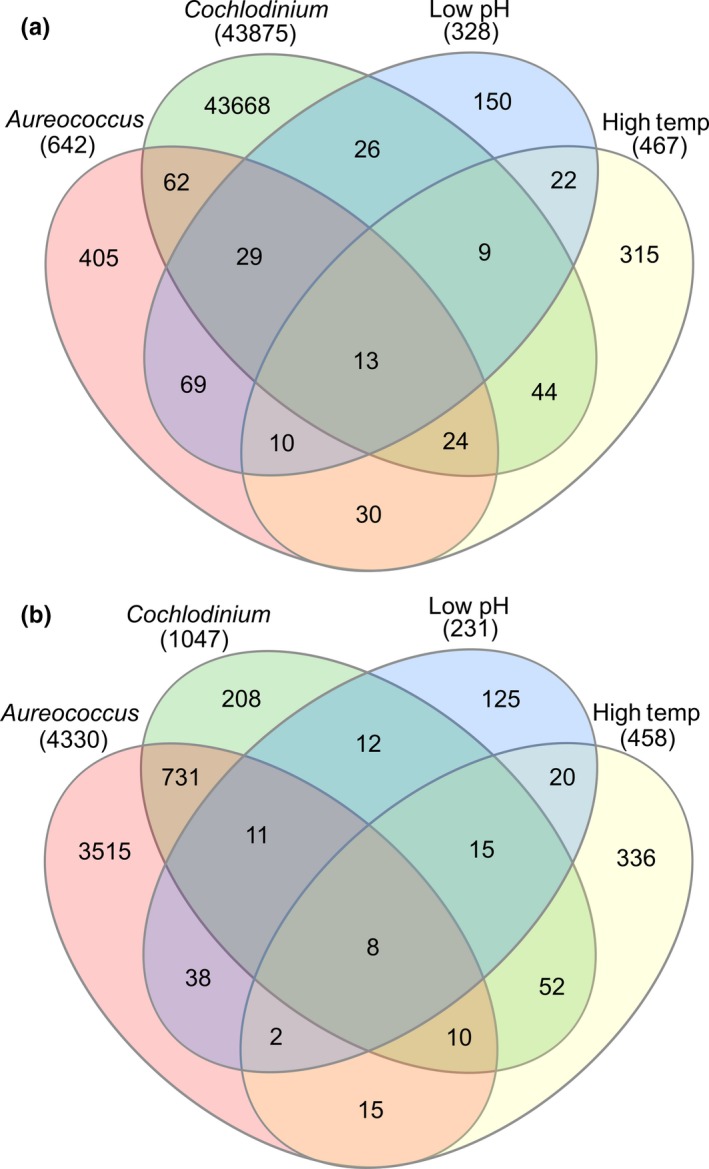
Venn diagram depicting the number of unique and shared transcripts between each treatment (a—transcripts observed to be at increased relative abundance; b—transcripts observed at lowered relative abundances)

A core of 21 genes was differentially (*padj* < 0.05) expressed by all stressor treatments (Figure 3). Of those, 13 had increased abundance, seven of which were annotated and found to encode reticulocyte‐binding proteins (reitculocyte‐binding protein 2 homolog a), organic cation transporters (isoform B), mitochondrial import inner membrane translocase (tim21), protein synthesis (tryptophan‐tRNA ligase), endoplasmic reticulum membrane proteins (subunit 10), multivesicular body (subunit 12B—vesicular trafficking), and a leucine‐rich repeat‐containing protein (Table 6). The remainder of transcripts (8) had decreased transcript abundance relative to the control (Figure 3) of which, two were annotated and identified as a tektin‐3 (cytoskeleton component) coding gene and a polybromo‐1 transcription factor (Table 6).

**Table 6:** Transcripts commonly up‐ (see rows 1–7) and down‐regulated (see rows 8 and 9) by scallop larvae in each larval treatment

	Sequence ID	Differential expression	Protein	Gene ID (Uni Prot)	Function
1	comp417773_c0_seq3	Up‐regulated	Reticulocyte‐binding protein 2 homolog a	PF13_0198	Reticulocyte function
2	comp426480_c0_seq10	Up‐regulated	organic cation transporter, isoform B	Orct2	Membrane transport
3	comp392847_c0_seq4	Up‐regulated	Mitochondrial import inner membrane translocase subunit Tim21	TIM21	Mitochondrial membrane transport
4	comp427123_c0_seq6	Up‐regulated	Tryptophan—tRNA ligase, mitochondrial	WARS2	Protein synthesis
5	comp425334_c0_seq7	Up‐regulated	ER membrane protein complex subunit 10	Emc10	ER membrane protein
6	comp423150_c0_seq3	Up‐regulated	Multivesicular body subunit 12B	MVB12B	Regulator of vesicular trafficking
7	comp420028_c0_seq14	Up‐regulated	Leucine‐rich repeat‐containing protein 59	LRRC59	Nuclear transport
8	comp414816_c1_seq5	Down‐regulated	Tektin‐3	TEKT3	Structural component of ciliary and flagellar microtubules
9	comp422207_c0_seq59	Down‐regulated	Protein polybromo‐1	PBRM1	Transcription factor

**Figure 4:**
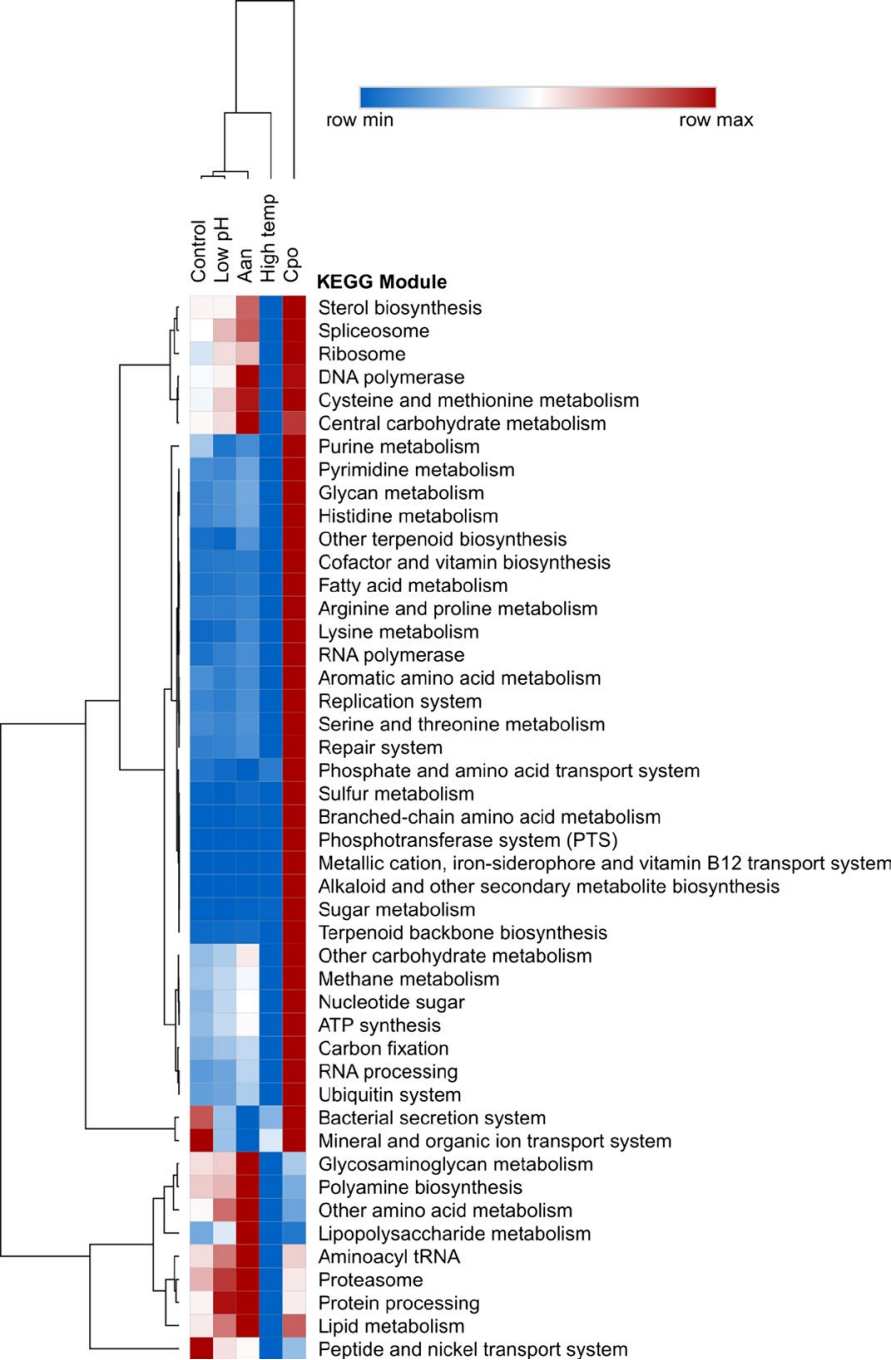
Hierarchically clustered heat map of normalized read counts for transcripts assigned KEGG ID's, binned by KEGG module for larvae within control, low pH, *A. aureococcus* (Aan), elevated temperature, and *C. polykrikoides* (Cpo) treatments (blue and red indicate minimum and maximum read count for each KEGG module, respectively)

Analyses of KEGG pathways revealed multiple biochemical pathways that were affected by exposure to each stressor with substantial differences in expression patterns observed between treatments (Figure 4). Larvae exposed to *C. polykrikoides* displayed the largest increase in transcript abundance (i.e., normalized read count) for many of the KEGG metabolic pathways relative to the other treatments (Figure 4). The weakest metabolic response (i.e., minimum normalized read count) was in the high temperature treatment where high‐abundance reads were only observed for KEGG pathways involved in biosynthesis of polyphenolic compounds and degradation of aromatic compounds (Figure 4). The control, low pH, and *A. anophagefferens* treatments elicited similar metabolic responses for many of the KEGG modules including those involved in purine/pyrimidine metabolism, fatty acid metabolism, RNA polymerase, sugar metabolism, and vitamin biosynthesis (Figure 4).

Exposure to either harmful algal species yielded the most intense responses among KEGG‐mapped pathways. Larvae exposed to *A. anophagefferens* displayed the greatest abundance of transcripts involved in the synthesis of DNA, carbohydrate metabolism, polyamine biosynthesis, cysteine and methionine metabolism, central carbohydrate metabolism, lipopolysaccharide metabolism, aminoacyl tRNA synthesis, proteosome complexes, protein processing, and lipid metabolism (Figure 4). Larvae exposed to *C. polykrikoides* exhibited the greatest number of transcripts associated protein synthesis (RNA polymerase, histidine, arginine, proline, lysine, aromatic amino acid metabolism etc.), cellular metabolism (ATP synthesis, sugar metabolism, other carbohydrate metabolism, glycan metabolism, and fatty acid metabolism), immune response (bacterial secretion), membrane transport (mineral and organic ions), and nucleic acid synthesis and repair (e.g., DNA & RNA polymerase, purine and pyrimidine metabolism, replication system, RNA processing, repair systems Figure 4). Compared to other treatments, larvae exposed to harmful algae (either species) exhibited greater expression of pathways associated with sterol synthesis, spliceosomes, ribosomal proteins, DNA polymerase, cysteine and methionine metabolism, and carbohydrate metabolism. Transcripts associated with these pathways were expressed at relatively lower abundances among the remaining treatments (Figure 4).

### 3.4 Targeted gene responses

**Figure 5:**
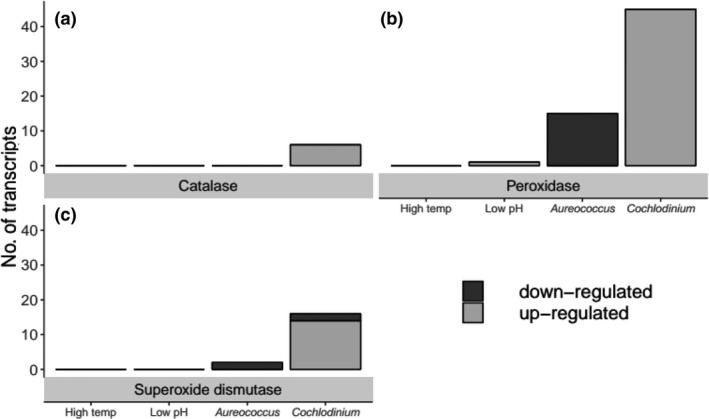
The number of significantly (*padj < *0.05) and differentially expressed unique transcripts corresponding to (a) catalase, (b) peroxidase, and (c) superoxide dismutase within each treatment

Several transcripts associated with generalized stress responses and/or self‐defense mechanisms were differentially expressed by larvae in each treatment but at varying levels of intensity. For example, larvae exposed to both harmful algal species responded by altering the expression of several transcripts associated with antioxidant defense mechanisms (Figure 5a–c). Specifically, larvae exposed to *C. polykrikoides *up‐regulated multiple transcripts associated with catalase, peroxidase, and superoxide dismutase whereas exposure to *A. anophagefferens* caused similar transcripts to be down‐regulated (Figure 5a–c). Within the acidification treatment only a single transcript corresponding to peroxidase (comp973279_c0_seq1; see Supporting Information Tables S3–S5) was observed to be differentially (*padj* < 0.05) expressed and there were no antioxidant transcripts differentially expressed by larvae exposed to thermal stress (Figure 5b).

**Figure 6:**
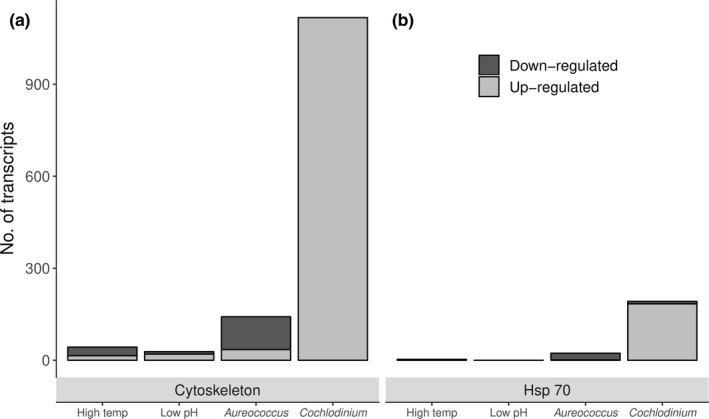
The number of significantly (*padj < *0.05) and differentially expressed unique transcripts corresponding to the (a) cytoskeleton and (b; Hsp 70) heat shock proteins within each treatment

A large number of transcripts (1,117) associated with the cytoskeleton were up‐regulated by larvae exposed to *C. polykrikoides *whereas the majority of those expressed by larvae within the *A. anophagefferens* treatment (107 of 142) were down‐regulated (Figure 6a). Fewer cytoskeletal transcripts were differentially expressed among larvae exposed to low pH and elevated temperature (28 and 43, respectively), but when exposed to low pH, larvae up‐regulated 20 cytoskeletal transcripts whereas when exposed to high temperature, larvae down‐regulated 28 cytoskeletal transcripts (Figure 6a). Similarly, 192 transcripts corresponding to heat shock proteins (e.g., Hsp 70, 74, and 90) were observed differentially expressed among *C. polykrikoides*‐exposed larvae with the majority (184) of those being up‐regulated (Figure 6b). Fewer heat shock protein transcripts were differentially expressed among *A. anophagefferens*‐exposed larvae, but the single transcript observed to be up‐regulated exhibited an increase in abundance by several orders of magnitude (Figure 6b; Table 2). Three Hsp transcripts were differentially expressed among larvae reared at elevated temperature, two of which were down‐regulated (comp422128_c0_seq5 & comp423081_c0_seq4; Supporting Information Tables S3–S5; Figure 6b) relative to control and one up‐regulated (comp415672_c1_seq; Suppl. Tables 3, 4, 5; Figure 6b).

**Figure 7:**
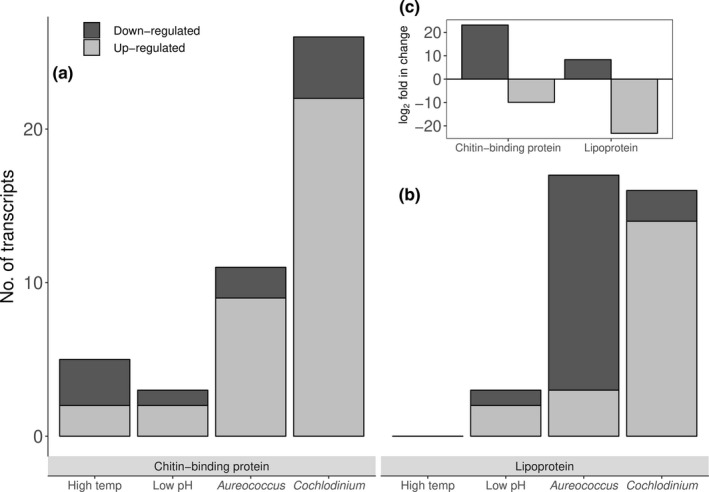
The number of significantly (*padj < *0.05) and differentially expressed unique transcripts associated with chitin‐binding (a) and lipoproteins (b; i.e., shell formation). Inset (c) depicts log2 fold change (relative to control treatments) in expression for shell growth transcripts expressed by acidification‐exposed larvae only

Transcripts corresponding to electron transport (e.g., oxidative phosphorylation) were also differentially expressed among larval treatments (Figure 7a–d). The expression of multiple transcripts encoding for cytochrome *c* oxidase, succinate dehydrogenase, malate dehydrogenase, and NADPH dehydrogenase was up‐regulated by larvae exposed to *C. polykrikoides *while fewer such transcripts were differentially expressed by larvae in the *A. anophagefferens* treatment (Figure 7a–d). Only one transcript associated with mitochondrial activity was differentially expressed within the elevated temperature treatment and was expressed at lower abundance relative to both harmful algal treatments (Figure 7a–d). No transcripts associated with electron transport were observed differentially expressed by larvae within the low pH treatment (Figure 7a–c).

**Figure 8:**
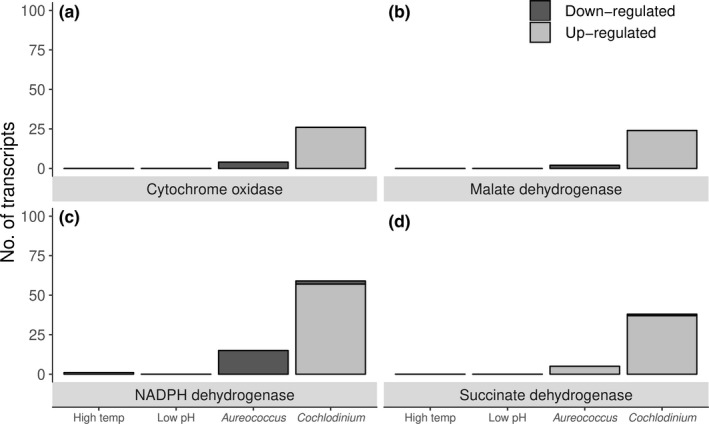
The number of significantly (*padj < *0.05) and differentially expressed unique transcripts corresponding to cytochrome *c* oxidase (a), malate dehydrogenase (b), NADPH dehydrogenase (c), and succinate dehydrogenase (d) within each treatment

With regard to genes associated with larval shell synthesis, chitin‐binding (e.g., protein obstructor‐E) and lipoproteins transcripts were differentially expressed by larvae in experimental treatments (Figure 8a,b; Schonitzer & Weiss, 2007; De Wit et al., 2018). Exposure to *C. polykrikoides *stimulated an up‐regulation of a large portion of differentially expressed transcripts associated with shell formation while exposure to *A. anophagefferens* caused a majority to be suppressed (Figure 8a). Mixed responses were observed among larvae exposed to thermal stress and acidification with larvae subjected to thermal stress exhibiting no differential expression of lipoproteins, but up‐regulated two (comp359259_c0_seq1 & comp415920_c1_seq5; see Supporting Information Tables S3–S5) and down‐regulated three (comp404974_c0_seq2, comp408153_c0_seq5, comp418944_c0_seq1; see Supporting Information Tables S3–S5) transcripts corresponding to chitin‐binding. Larvae subjected to acidification exhibited significantly higher levels of two chitin‐binding transcripts and significantly lower levels for another. Large differences, however, were observed in the magnitude of expression between the two transcripts whereby the abundance of chitin‐binding transcripts in acidification treatments was increased by several orders of magnitude (e.g., comp394498_c0_seq1 & comp382237_c0_seq1; See Supporting Information Tables S3–S5; Figure 7c—inset) and the single lipoprotein transcript observed to be down‐regulated was lowered by several orders of magnitude (comp 427695_c0_seq1; See Supporting Information Tables S3–S5; Figure 7c—inset).

## 4 DISCUSSION

Bay scallop populations along the eastern US have been in decline during the past four decades, trends partly driven by estuarine stressors that are predicted to intensify this century. Here, we documented the transcriptomic responses of larval bay scallops exposed to acidification, warming, and harmful algae revealing unique physiological and biochemical pathways affected in larvae and potential adaptive mechanisms employed by larval scallops to cope with stress. The multiple stressors included here allowed for direct comparison of unique and shared transcriptomic responses among larvae exposed to varying abiotic and biotic stressors common to marine habitats within the geographic range of the bay scallop.

Universal stressor responses of bay scallops exposed to coastal zone stressors included the down‐regulation of transcripts associated with the transcription factor, protein polybromo‐1 suggesting stressors either inhibit the expression/transcription of these proteins or that scallop larvae divest energy associated with these ATP‐dependent (Porter & Dykhuizen, 2017) pathways into other processes in response to stress. Other responses inhibited by all stressors included transcripts associated with tektin‐3, components of ciliary and flagellar microtubules, again suggesting that this is a particularly sensitive or expendable pathway and that energy associated with this process is simply diverted to others in response to an environmental stressor. Transcripts that became more abundant following exposure to all stressors included those involved with reticulocyte‐binding (protein 2 homolog a), membrane transport (organic cation transporter, isoform B), cellular metabolism (mitochondrial import inner membrane translocase subunit TIM21), protein synthesis and transport (tryptophan‐tRNA ligase, ER membrane protein complex subunit 12B, multivesicular body subunit 12 B), and nuclear transport (leucine‐rich repeat‐containing protein 59; see Table 2). Collectively, the overexpression of these transcripts suggests that energy production mechanisms are up‐regulated in response to stressors, allowing for increased synthesis of certain proteins, presumably those involved with a generalized stress response.

### 4.1 Larval responses to elevated temperature

Unlike previous investigations challenging larval scallops to thermal stress (Talmage & Gobler, 2011), survival within the elevated temperature treatment was only slightly reduced and not significantly relative to the control, perhaps in part, due to the short duration of this experiment (10 days). Further, while the optimum temperature for the normal development of bay scallop larvae is 24°C (Shumway & Parsons, 2011), bay scallops have a geographic range that extends south to the Gulf of Mexico (Blake & Shumway, 2006; Clark, 1965) where larvae may commonly experience extended periods of 30°C. Transcriptomic responses of larval scallops exposed to thermal stress (~30°) were relatively modest with only a few transcripts associated with known biochemical pathways (e.g., degradation of aromatic compounds and synthesis of polyphenolic compounds) being observed at greater abundance than in the other treatments. The expression of transcripts associated with generalized stress response (e.g., Hsp 70 and cytoskeleton‐associated proteins), antioxidant responses, energy capture, and shell growth were less intense (i.e., log_2 _fold change) than responses by larvae exposed to harmful algae (see Supporting Information Tables S3–S5). Collectively, these findings demonstrate that among stressors included in the current study, elevated temperature was the least impactful at the transcriptional level, but when combined with others stressors (e.g., hypoxia, low pH, and exposure to harmful algae) more severe outcomes may occur (Pörtner, 2008, 2010). Further, it is likely that more extreme temperatures would have elicited a broader and/or stronger response.

### 4.2 Larval responses to low pH

Similar to previous studies involving larval scallops exposed to acidification (Talmage & Gobler, 2009,2010), significant reductions in survival were observed during this study (Figure 1). Beyond survival, our findings revealed specific pathways invoked by larval scallops exposed to acidification. For example, scallops reared at low pH increased the expression of transcripts associated with antioxidant defense (e.g., peroxidase) and heat shock proteins. Similar responses by *C. virginica* have been reported (Goncalves et al., 2016; Tomanek, Zuzow, Ivanina, Beniash, & Sokolova, 2011) whereby acidification‐exposed larvae up‐regulated several antioxidant enzymes and transcripts associated with generalized stress responses suggesting that, beyond inhibiting early‐larval calcification (Gobler & Talmage, 2013; Waldbusser et al., 2013), acidification causes substantial oxidative stress (Goncalves et al., 2016; Tomanek et al., 2011). Alternatively, overexpression of these enzymes may arise indirectly via an elevated metabolic rate as evidenced by the up‐regulation of genes associated with cellular metabolism (e.g., cytochrome *c* oxidase; *detailed above*). Further, Waldbusser et al. (2015) documented that exposure to acidification induced elevated respiration in larval shellfish suggesting an increased abundance of antioxidants may be a response to metabolic processes that are simulated by high CO_2_ conditions (Goncalves et al., 2016). Larvae within the low pH treatment also differentially expressed 20 transcripts associated with cytoskeleton production, responses that may also be related to oxidative stress. Cytoskeleton‐associated microfilaments are a common target of reactive oxygen species (Dalle‐Donne, 2001) and/or are an additional means by which cells defend themselves against oxidative stress (Dalle‐Donne, Giustarini, Rossi, Colombo, & Milzani, 2003; Dalle‐Donne, Rossi, Giustarini, Colombo, & Milzani, 2003). While the overall number of transcripts associated with the cytoskeleton that were differentially expressed by larvae exposed to acidification was relatively low, the magnitude (i.e., log_2_‐fold change) of expression for some transcripts (e.g., comp405629_c0_seq11 & comp425670_c0_seq4; see Supporting Information Tables S3–S5) were increased by several orders of magnitude suggesting that some of these cytoskeleton transcripts may also be involved in shell synthesis which is compromised by acidification (Waldbusser et al., 2015).

Beyond antioxidant defenses, transcripts associated with early‐larval shell growth were differentially expressed among larvae exposed to acidification. Similar to prior studies involving larval bivalves subjected to acidification (De Wit et al., 2018), transcripts associated with early‐shell growth were suppressed. Specifically, two transcripts involved in early‐shell synthesis (De Wit et al., 2018) were found to be up‐regulated, however the single transcript that was suppressed by acidification was down‐regulated by several orders of magnitude more than those that were up‐regulated. Conversely, as discussed above, the up‐regulation of certain cytoskeletal‐associated transcripts may be related to early‐shell formation by larval scallops. Chitin‐binding proteins, an essential component for early‐shell and nacre formation (Schonitzer & Weiss, 2007; Weiss, Schönitzer, Eichner, & Sumper, 2006), were among the most up‐regulated transcripts (comp394489_c0_seq1 & comp382237_c0_seq1; See Supporting Information Tables S3–S5; Figure 8c—inset) by larvae within the acidification treatment and may be a mechanism by which larval scallops increase the rate of shell formation in low pH environments (De Wit et al., 2018). Further, the concomitant differential expression of transcripts associated with cellular metabolism and protein synthesis (see Figure 5) by larval scallops exposed to acidification is likely required to support broad cellular defenses and shell growth.

### 4.3 Larval responses to *C. polykrikoides*

In recent decades, *C. polykrikoides *blooms have become increasingly widespread across the Northern Hemisphere (Kudela & Gobler, 2012). Climate change, and ocean warming in particular, have enabled and expansion of blooms that grow faster and persist longer (Griffith, A.W., Doherty, O.M., and Gobler, C.J. 2019). Blooms of *C. polykrikoides* are capable of causing large‐scale die‐offs of marine organisms, especially among caged and aquacultured organisms (Griffith, Shumway, & Gobler, 2018; Kim, 1998; Kim, Lee, & An, 1997). While multiple harmful modes of action related to this alga have been proposed (Kim, Lee, Lee, Kim, & Jung, 1999; Kim et al., 2002; Onoue & Nozawa, 1989), the majority of evidence suggests lethal effects are associated with the production of ROS (Kim et al., 1999; Tang & Gobler, 2009a,2009b), compounds capable of causing severe oxidative damage (Miller, Suzuki, Ciftci‐Yilmaz, & Mittler, 2010; Wise, 1995; Yu, 1994). Previous studies have demonstrated that the enzymes catalase and peroxidase can mitigate lethal effects of this alga (Griffith & Gobler, 2016; Tang & Gobler, 2009a,2009b). Consistent with these findings, multiple antioxidant‐associated transcripts were differentially expressed by larvae exposed to *C. polykrikoides* and at levels significantly greater than those exhibited by larvae in other treatments. Specifically, the number of differentially expressed transcripts corresponding to catalase and superoxide dismutase activity were two‐ to ten‐fold greater among larvae exposed to *C. polykrikoides*. The majority of transcripts associated with peroxidase were either not differentially expressed or were observed at lowered levels than in other treatments whereas 45 unique transcripts were differentially expressed at higher levels by larvae exposed to *C. polykrikoides*. In addition to a greater variety of unique transcripts, levels of expression (i.e., log_2_‐fold change) of specific transcripts were several orders of magnitude greater than those of larvae in the remaining treatments (see Supporting Information Table S1). Collectively, findings suggesting these enzymes are overexpressed in response to ROS exuded by *C. polykrikoides* and/or are a generalized stress response to ROS as a consequence of heightened metabolism in larvae (Hégaret et al., 2011).

Differential expression of cytoskeleton‐associated transcripts by larvae exposed to *C. polykrikoides *(e.g., 1,117 transcripts) exceeded levels exhibited by larvae in all other treatments. The cytoskeleton, and associated proteins, are integral components of eukaryotic cells governing cellular movement, cell division/growth, translation, and cell signaling (Chuong et al., 2004; Davies, Fillingham, & Abe, 1996), as well as preventing oxidative damage (Dalle‐Donne, Giustarini, et al., 2003; Dalle‐Donne, Rossi, et al., 2003). Hence, enhanced expression of numerous cytoskeleton‐associated transcripts may be an indication that these cellular components are vulnerable and/or a common target of ROS or that these transcripts are broadly expressed by larval scallops to defend themselves against externally or internally sourced ROS (Dalle‐Donne, Giustarini, et al., 2003; Dalle‐Donne, Rossi, et al., 2003).

Beyond antioxidant‐associated genes, other transcripts expressed at greater abundances among *C. polykrikoides*‐exposed larvae include those associated with a generalized stress response. Specifically, 184 transcripts associated with Hsp (e.g., Hsp 70, 74, and 90; see Supporting Information Tables S3–S5) were expressed at greater abundances within *C. polykrikoides* treatments than in all other treatments including larvae subjected to thermal stress. The expression of Hsp transcripts were several orders of magnitude greater among *C. polykrikoides*‐exposed larvae than levels of the few transcripts differentially expressed by larvae exposed to brown tide, acidification, and thermal stress (see Supporting Information Tables S3–S5). Prior work indicates Hsp are expressed by a wide range of organisms in response to numerous stressors/toxicants (Hofmann, 2005; Kiang, 1998; Kim, 1998). Heat shock proteins within bay scallop larvae exposed to *C. polykrikoides *are likely indicative of a generalized defense response and may be expressed to prevent the damage of and/or promote the repair of proteins subjected to ROS (Gorman, Heavey, Creagh, Cotter, & Samali, 1999; Hofmann, 2005).

In addition to generalized responses, larvae exposed to *C. polykrikoides* stimulated the expression of several transcripts associated with electron transport (e.g., cytochrome *c* oxidase, malate dehydrogenase, NADPH dehydrogenase, and succinate dehydrogenase). Elevated levels of these transcripts are presumably required to support broad responses employed by larvae to defend against *C. polykrikoides,* specifically those involved in preventing oxidative damage. Given the number of unique transcripts differentially expressed and the magnitude of expression, scallop larvae presumably invest significant amounts of energy into cellular defense when exposed to *C. polykrikoides*. Even under ideal conditions, large amounts of energy are required by bivalve larvae to precipitate their initial shell (e.g., <48 hr; Waldbusser et al., 2013; Frieder, Applebaum, Pan, Hedgecock, & Manahan, 2017). Hence, responses elicited by *C. polykrikoides* may exhaust energy reserves within rapidly developing larvae contributing to their mortality. Beyond direct toxicity and the ability to deplete energy reserves within larval bivalves, *C. polykrikoides* may adversely affect larval shellfish via food‐limitation, as cells may be too large to be efficiently captured and ingested by early‐stage bivalve larvae (Helm et al., 2004; Raby et al., 1997). Post‐hatch (i.e., after prodissoconch I shell formation) shellfish larvae are reliant upon exogenous food sources (Helm et al., 2004; Waldbusser et al., 2015) and bivalve larvae (e.g., veliger‐stage; 185–260 µm), including bay scallops, readily ingest algal particles from 5–15 µm in length whereas the capture and ingestion of larger particles is significantly lower (Raby et al., 1997). *C. polykrikoides* cells are considerably larger (~35 µm long; Gobler et al., 2008) than particles optimally ingested by shellfish larvae and bay scallop larvae, at the time of harvest (48 hr), were smaller (~100 µm) than those included in other studies (see Raby et al., 1997), thus less likely to ingest cells larger than 15 µm (Helm et al., 2004). Hence, beyond direct toxicity via the exudation of toxicants (i.e., ROS) and the subsequent diversion of energy from homeostatic processes, *C. polykrikoides* may indirectly impact larval shellfish via food‐limitation which may, in part, contribute to the broad transcriptomic responses observed among exposed larvae.

### 4.4 Larval responses to *A. anophagefferens*

Numerous studies have reported on the toxicity of *A. anophagefferens *to bivalve shellfish (Bricelj & MacQuarrie, 2007; Gallager, Stoecker, & Monica, 1989; Gobler & Sunda, 2012; Harke, Gobler, & Shumway, 2011; Tracey, 1988) including larval bay scallops (Gallager et al., 1989), findings consistent with those observed here as final (10 day) survival of larvae exposed to *A. anophagefferens *was <1%. Exposure to *A. anophagefferens *resulted in an order of magnitude fewer differentially expressed transcripts compared to *C. polykrikoides* exposure but also an order of magnitude larger response than those elicited by acidification and thermal stress. Further, while scallop larvae exposed to *C. polykrikoides *displayed broad, up‐regulation of multiple pathways, exposure to *A. anophagefferens* substantially reduced the abundance of many transcripts. Specifically, antioxidant defenses, other generalized stress responses (e.g., heat shock and cytoskeleton proteins), and transcripts associated with electron transport were all largely down‐regulated. These responses are consistent with prior reports suggesting that exposure to *A. anophagefferens *interferes with chemosensory function (Gallager et al., 1989) and causes physiological incapacitation of bivalve larvae (Bricelj & MacQuarrie, 2007) as well as highlights differing harmful mechanisms between the two HABs. While exposure to *C. polykrikoides* stimulated broad increases in the expression of numerous biochemical and physiological pathways, at levels that were likely not energetically sustainable for developing larvae, exposure to *A. anophagefferens *causes a down‐regulation of defense mechanisms. The differing survival trends between the two harmful algae (rapid mortality from *C. polykrikoides, *slower mortality from *A. anophagefferens*), combined with the transcriptomic response provides insight into the associated mortality mechanisms. The ROS‐like toxicants released by *C. polykrikoides* were seemingly a more acute threat to larvae, yielding an intense transcriptomic response and rapid mortality after 48 hr. In contrast, suppression of multiple biochemical pathways among scallops exposed to *A. anophagefference* may have been of benefit in the short‐term, as survival after 48 hr did not differ from the control. Over 10 days, however, these responses were not sustainable, as the 10‐day survival rate for both harmful algae treatments was <3% and significantly lower than all other treatments.

Similar to *C. polykrikoides*, multiple modes of action have been proposed for *A. anophagefferens *including direct toxicity via the exudation of extracellular toxicants and food‐limitation (Bricelj & MacQuarrie, 2007; Cosper et al., 1987; Gallager et al., 1989). *A. anophagefferens *cells (~2 µm) are poorly retained by scallop larvae and also inhibit the ingestion of co‐occurring, non‐toxic algal species (Gallager et al., 1989), thus leading to a poor nutritive state. Multiple harmful modes of action (e.g., food‐limitation and production of toxicants) between the two harmful algal species may, in part, explain the large differences in the overall differential expression between the biotic and abiotic stressors included in this study. Given that both harmful algae are sources of poor nutrition and the near‐opposite transcriptomic responses by larvae between the two treatments, starvation may not have been the primary driver of gene expression between the two treatments. Hence, the intense and acute toxicity of *C. polykrikoides* led to the mass up‐regulation of genes to repair and defend, whereas the noxious but less intense toxic properties of *A. anophagefferens* led to a mass down‐regulation of biochemical pathways that protected larvae in the short‐, but not long‐term.

### 4.5 Bay scallop population ecology

Bay scallop populations along the eastern US have encountered numerous challenges in recent decades with overharvest (Barber & Davis, 1997), habitat loss (Orth et al., 2006; Serveiss et al., 2004), and recurring harmful algal blooms (Cosper et al., 1987; Summerson & Peterson, 1990) contributing to a 97% decline in landings since the 1980s (NOAA Fisheries Landings 1980–2016; www.noaa.gov) and population‐level declines in areas where bay scallop fisheries were once significant (Tettelbach et al., 2013). While some bay scallop restoration efforts have exhibited success (Tettelbach et al., 2015), findings presented here demonstrate that the presence of multiple stressors may complicate future restoration. As carbon emissions continue to warm and acidify coastal ecosystems, the associated adverse effects on bay scallop populations may intensify. As anthropogenic activities continue to establish conditions along coastal zones that are more permissive for the growth and persistence of multiple species of harmful algae (Gobler et al., 2017; Heisler et al., 2008), further population‐level declines among bay scallops are likely. Given that realistic levels of both harmful algal species and climate change stressors were used in the current study, the more intense mortality and transcriptomic responses to harmful algae compared to climate change stressors by the scallop larvae suggest that HABs may be a more pressing threat to bay scallops in the near‐future.

## 5 CONCLUSION

The unique responses of bay scallop larvae exposed to multiple coastal zone stressors presented here provide a novel assessment of how biochemical and physiological pathways are differentially affected by these stressors and provide mechanistic insight into their harmful effects. A core set of transcripts elicited by all stressors indicate biochemical pathways associated with energy production and stress are broadly implemented by shellfish larvae. Exposure to HABs elicited the largest responses in terms of both the number of differentially expressed transcripts and the level of expression. While exposure to *C. polykrikoides* elicited the up‐regulation of multiple biochemical pathways meant to mitigate immediate oxidative damage and generalized stress, exposure to *A. anophagefferens* suppressed cellular defense mechanisms and energy production pathways likely contributing to delayed mortality. Exposure to low pH and elevated temperature elicited more modest responses with regard to the number of transcripts differentially expressed by scallop larvae, although transcripts associated with shell formation were responsive to acidification. Differences in transcriptomic profiles and survival of bay scallop larvae between abiotic and biotic stressor treatments may, in part, be a result of multiple modes of action (e.g., direct toxicity and food‐limitation) associated with exposure to harmful algae. Collectively, results indicate that future global change stressors will impact marine life in numerous ways that will threaten to bay scallop populations existing within at‐risk locales.

## CONFLICT OF INTEREST

None declared.

## AUTHOR CONTRIBUTIONS

ED performed laboratory‐based exposures. AWG, MJH, and DLB analyzed sequence data. AWG, MJH, and CJG wrote and edited the manuscript.

## Data Availability

All data are available upon request. Sequence data are deposited in the Sequence Read Archive through the National Center for Biotechnology Information under accession no. SRP159112.
