# Supplementary material for: The harmful algae, Cochlodinium polykrikoides and Aureococcus anophagefferens, elicit stronger transcriptomic and mortality response in larval bivalves (Argopecten irradians) than climate change stressors
Source: Ecol Evol. 2019 Apr 5;9(8):4931–48. doi: 10.1002/ece3.5100 (PMC6476759; doi:10.1002/ece3.5100)
Supplement: Supplementary file 6 [file ECE3-9-4931-s006.docx]

**Supplemental Table Captions**

Supplemental table 1**.** Summary of sequencing data of *Argopectin irradians* transcriptome. The “Trinity” column depicts summary of initial Trinity assembly while the “Trinity98” column depicts the summary of the assembly after clustering with CD-Hit-EST at 98% identity.

Supplemental table 2. Alignment results from RSEM with Bowtie 2.

Supplemental table 3. Complete list of contigs considered for differential expression analysis detailing annotations and raw read counts for each treatment. Where no annotation was found a "." is presented.

Supplemental table 4. Differential expression results relative to the Control for each Treatment.

Supplemental table 5. Complete list of contigs differenatially expressed and considered for targeted gene responses
